# Survival Kinetics of Starving Bacteria Is Biphasic and Density-Dependent

**DOI:** 10.1371/journal.pcbi.1004198

**Published:** 2015-04-02

**Authors:** Andy Phaiboun, Yiming Zhang, Boryung Park, Minsu Kim

**Affiliations:** 1 Department of Physics, Emory University, Atlanta, Georgia, United States of America; 2 Graduate Division of Biological and Biomedical Sciences, Emory University, Atlanta, Georgia, United States of America; Georgia Tech, UNITED STATES

## Abstract

In the lifecycle of microorganisms, prolonged starvation is prevalent and sustaining life during starvation periods is a vital task. In the literature, it is commonly assumed that survival kinetics of starving microbes follows exponential decay. This assumption, however, has not been rigorously tested. Currently, it is not clear under what circumstances this assumption is true. Also, it is not known when such survival kinetics deviates from exponential decay and if it deviates, what underlying mechanisms for the deviation are. Here, to address these issues, we quantitatively characterized dynamics of survival and death of starving *E*. *coli* cells. The results show that the assumption – starving cells die exponentially – is true only at high cell density. At low density, starving cells *persevere* for extended periods of time, before dying rapidly exponentially. Detailed analyses show intriguing quantitative characteristics of the *density-dependent and biphasic* survival kinetics, including that the period of the perseverance is inversely proportional to cell density. These characteristics further lead us to identification of key underlying processes relevant for the perseverance of starving cells. Then, using mathematical modeling, we show how these processes contribute to the density-dependent and biphasic survival kinetics observed. Importantly, our model reveals a thrifty strategy employed by bacteria, by which upon sensing impending depletion of a substrate, the limiting substrate is conserved and utilized later during starvation to delay cell death. These findings advance quantitative understanding of survival of microbes in oligotrophic environments and facilitate quantitative analysis and prediction of microbial dynamics in nature. Furthermore, they prompt revision of previous models used to analyze and predict population dynamics of microbes.

## Author Summary

Long periods of starvation are common in the lifecycle of microorganisms. Textbooks routinely describe that during starvation periods, cells die at a constant rate, i.e., exponential decay. The exponential decay of cell survival has been commonly assumed in the literature to analyze and predict population dynamics of microbes. Here, we show that this assumption is true only at high cell density. At low cell density, cells can persevere for extended periods of time, before dying at a constant rate. Quantitatively analyzing the kinetics, we uncover mathematical formulas governing the density-dependent, biphasic decay of cell survival. Using mathematical modeling, we further reveal key underlying processes responsible for the perseverance. Our model highlights a thrifty strategy of bacteria; upon sensing impending starvation, small amounts of nutrients are conserved and used to persevere during starvation periods. In addition to advancing our fundamental understanding of physiology of bacteria in nature, our study will facilitate the analysis and prediction of microbial dynamics in nature. We expect that our findings will have broad impacts. For example, our findings can be used to accurately predict how pathogens survive in natural environments, which will lead to better public health policies.

## Introduction

Under favorable growth conditions, microorganisms can grow rapidly. For example, *E*. *coli* cells can grow as fast as ∼ 20 min per doubling under ideal growth conditions. If this rate continues, a single *E*. *coli* bacterium can generate the mass of the earth in *a couple of days*. Clearly exponential growth cannot be sustained infinitely. Eventually, nutrients required for cell growth will be depleted and cells will be subject to long periods of starvation. Indeed, a survey suggests that ecosystems are dominated by starving microbes [1].

Due to their dominance, understanding *quantitatively* how starving microbes live and die is of great interest in various fields of microbiology, ranging from analyzing microbial population dynamics in soils to predicting the number of microbes in freshwater. However, our quantitative understanding of survival kinetics of starving microbes is poor. In textbooks, survival kinetics has been commonly assumed as simple first order kinetics, i.e., exponential decay [2,3]. In the literature, this assumption has been widely used as a basis for analyzing and predicting microbial population dynamics, e.g., see [4–6]. This assumption, however, has not been rigorously tested. Currently, it is not clear under what circumstances this assumption is true. Also, it is not known when such survival kinetics deviates from exponential decay and if it deviates, what underlying mechanisms for the deviation are.

A large body of studies exists that characterizes starvation response at the molecular level; for example, see [7–10] for complex signaling pathways and gene regulations in response to starvation in proteobacteria. However, our molecular-level knowledge is still far from complete even for model systems such as *E*. *coli*. Also, much of our knowledge is qualitative and it is not clear to what degree these molecular processes affect cell survival. Thus, our molecular-level knowledge has not contributed to quantitative understanding of survival kinetics of starving cells.

In recent years, quantitative and phenomenological characterization of cellular processes proved to be a powerful approach for deeper understanding of complex biological systems, e.g., see [11–15]. In particular, it was shown that despite complex underlying molecular interactions, simple phenomenological laws governing cellular-level behaviors can exist and such laws greatly facilitate deeper understanding of underlying mechanisms [16].

In this work, using such phenomenology-to-mechanism approach, we rigorously characterized cell survival under starvation using *E*. *coli* as a model system. We show that survival kinetics of starving *E*. *coli* is biphasic and cell-density-dependent. Quantitative analyses reveal simple quantitative formulas governing the patterns, e.g., the first and second kinetics are well described by exp(-*t* ^2^) and exp(-*t*) respectively, and the duration of the first kinetics is inversely proportional to cell density. (The results show that the previous assumption—exponential decay of survival of starving cells—is true only at very high cell density.) Next, using this knowledge as a guide, we identified key underlying processes for cell survival. Using mathematical modeling, we showed how these processes contribute to the intricate survival patterns observed.

## Results and Discussion

### Survival of starving cells is cell-density-dependent and biphasic

Cells were grown in minimal media with glycerol as the sole carbon source (see Materials and methods). As cells grow, glycerol is consumed and eventually exhausted, leading to the cessation of growth; we provided low enough amounts of glycerol to ensure that the growth is arrested as a result of the exhaustion of glycerol, not by other nutrient sources (S1 Fig; see Materials and methods for the exact glycerol concentrations used). The cultures containing different amounts of glycerol in the medium initially result in different saturating densities of cells at the onset of the growth arrest (S1A Fig). The onset of growth arrest defines the time zero (S1B Fig). Afterwards, the number of colony-forming units, *N*_CFU_, was determined at various time points using a standard plate count method. We define the cells that grow on the agar plates and form colonies as viable.

**Fig 1:**
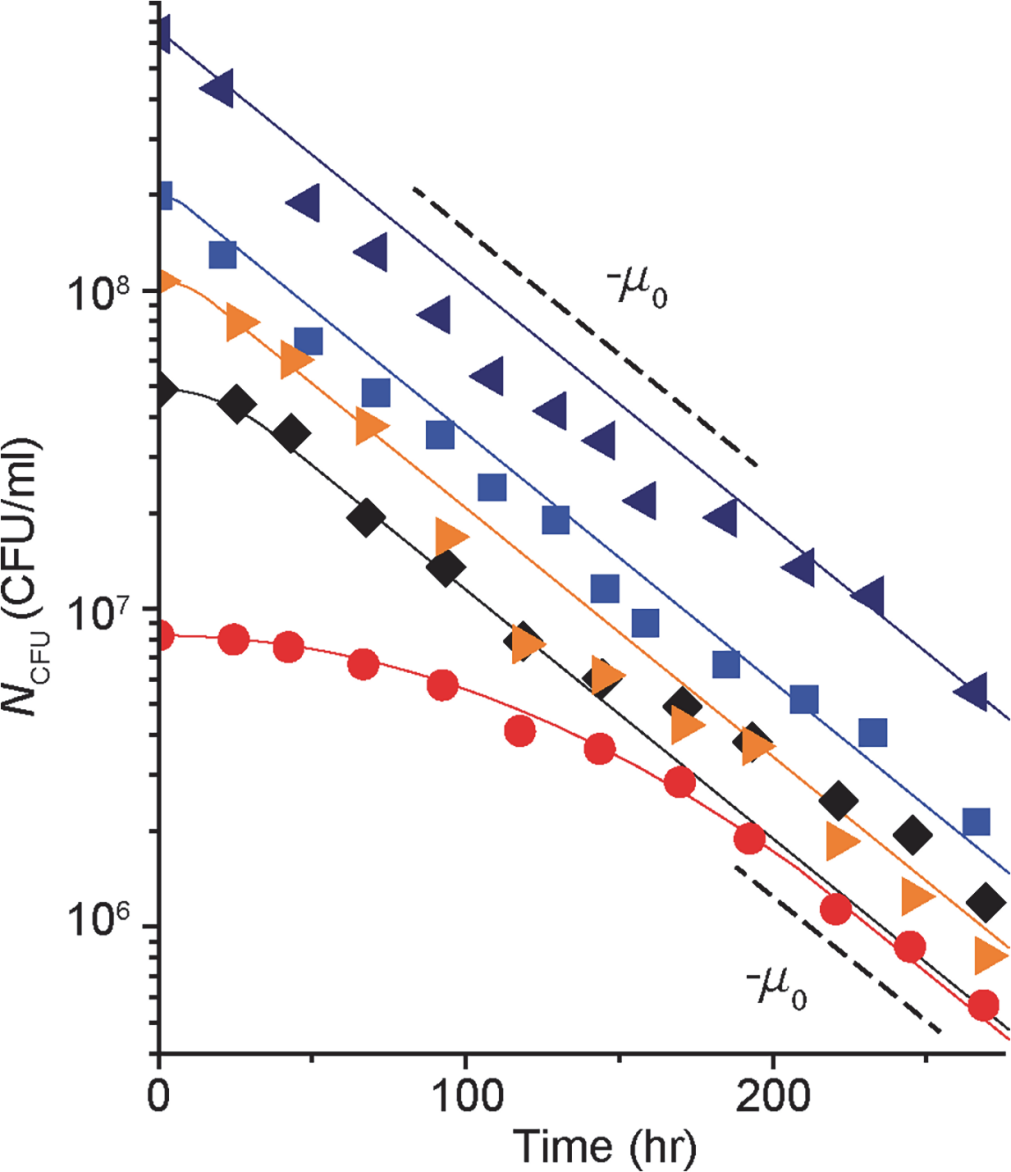
Temporal survival kinetics of starving *E*. *coli* cells. The number of colony-forming-unit (*N*_CFU_) of glycerol-depleted cultures is plotted over time in a semi-log graph. Different symbols indicate different cell densities at the onset of growth arrest. For clarity, *N*_CFU_ of five cultures (with different cell densities) is plotted. See S2 Fig for the complete set of data (also see S8 Fig for the reproducibility of the data). The dashed lines are plotted for a visual guide. The lines have the slope of −*μ*_0_ (= −0.018 hr ^-1^). For the cultures whose densities are higher than ∼10^8^ cells/ml (navy left triangles and blue squares), *N*_CFU_ follows single phase exponential decay with the rate of −*μ*_0_. In the cultures with lower densities, however, we see biphasic kinetics (black diamonds and red circles). Initially, *N*_CFU_ decreases gradually (the first phase), and eventually decreases exponentially at the rate of −*μ*_0_ (the second phase). The solid lines show the fits of our model (discussed further below in the text).

The temporal kinetics of *N*_CFU_ in glycerol-exhausted cultures with 5 different cell densities is plotted in Fig. 1 (see S2 Fig for the kinetics of other cell densities). For the cultures whose densities are higher than ∼10^8^ cells/ml, *N*_CFU_ follows a single phase exponential decay. The black dashed lines are plotted for a visual guide and its slope, −*μ*_0_ (= −0.018 hr ^-1^), corresponds to the rate of cell death in these cultures. Note that *N*_CFU_ of starving wild-type *E*. *coli* cells reported previously in the literature can be well approximated by a single-phase exponential decay [17–19].

In the cultures with lower densities, however, we see biphasic kinetics of *N*_CFU_ (see black diamonds and red circles in Fig. 1 and purple hexagons and green triangles in S2 Fig); *N*_CFU_ gradually decreases initially (the first phase) and eventually decreases exponentially at the rate of −*μ*_0_ (the second phase). The period of the first phase becomes more pronounced at lower cell density, prolonging cell survival. When we repeated this experiment using other carbon sources or using a different *E*. *coli* strain, we observed similar density-dependent biphasic kinetics of *N*_CFU_ (S3 Fig and S4 Fig).

Previously, it was shown that cultures starved of nutrients for a long time yield mutants with increased fitness, called the growth advantage in stationary phase (GASP) phenotype [20,21]. The appearance of GASP mutants results in visible change in *N*_CFU_; *N*_CFU_ initially decreasing at a constant rate reaches a plateau when GASP mutants appear. The timing of appearance of GASP mutants depends on the types of media and bacterial strains used [21]; for example, in Luria-Bertani (LB) media, they appeared within several days of growth cessation [21] while in other starvation experiments using minimal media, they appeared after ~ 30 days of starvation [22]. In our experiment, using minimal media, we performed experiments for ~ 12 days. During this time, we did not observe such transition in *N*_CFU_ (i.e., from a rapid decrease to a plateau); see Fig. 1. This strongly suggests that GASP mutants have not appeared during our experiments. Also, when we repeated the experiment using cells from single colonies obtained from the cultures starved for 12 days, we observed that *N*_CFU_ of these cells decreases very similarly as *N*_CFU_ shown in Fig. 1 (S5 Fig). Taken together, we conclude that GASP mutants have not appeared and played no role in the survival kinetics observed in our experiments.

### The density-dependent, biphasic kinetics of cell survival is not due to extracellular signaling

It is well known that microorganisms use extracellular signaling to sense the density of the populations and coordinate their behaviors accordingly [23]; they secrete extracellular signals and such signals accumulate in the medium, allowing cells to sense the density and regulate their behaviors accordingly. The cost and benefit of such extracellular signaling has been recently demonstrated quantitatively [24]. It is possible that the density-dependent kinetics of survival and death observed in Fig. 1 could be also mediated by such extracellular signaling; such (potential) signals may accumulate to high levels in high cell-density cultures, or in low cell-density cultures at a later time, triggering a rapid, exponential decay of *N*_CFU_. Note that the second case requires the signals to be stable at least for ∼100 hrs (see Fig. 1). To test this possibility, we repeated the experiment above using media designed to remove or concentrate these potential secreted factors, as described below.

**Fig 2:**
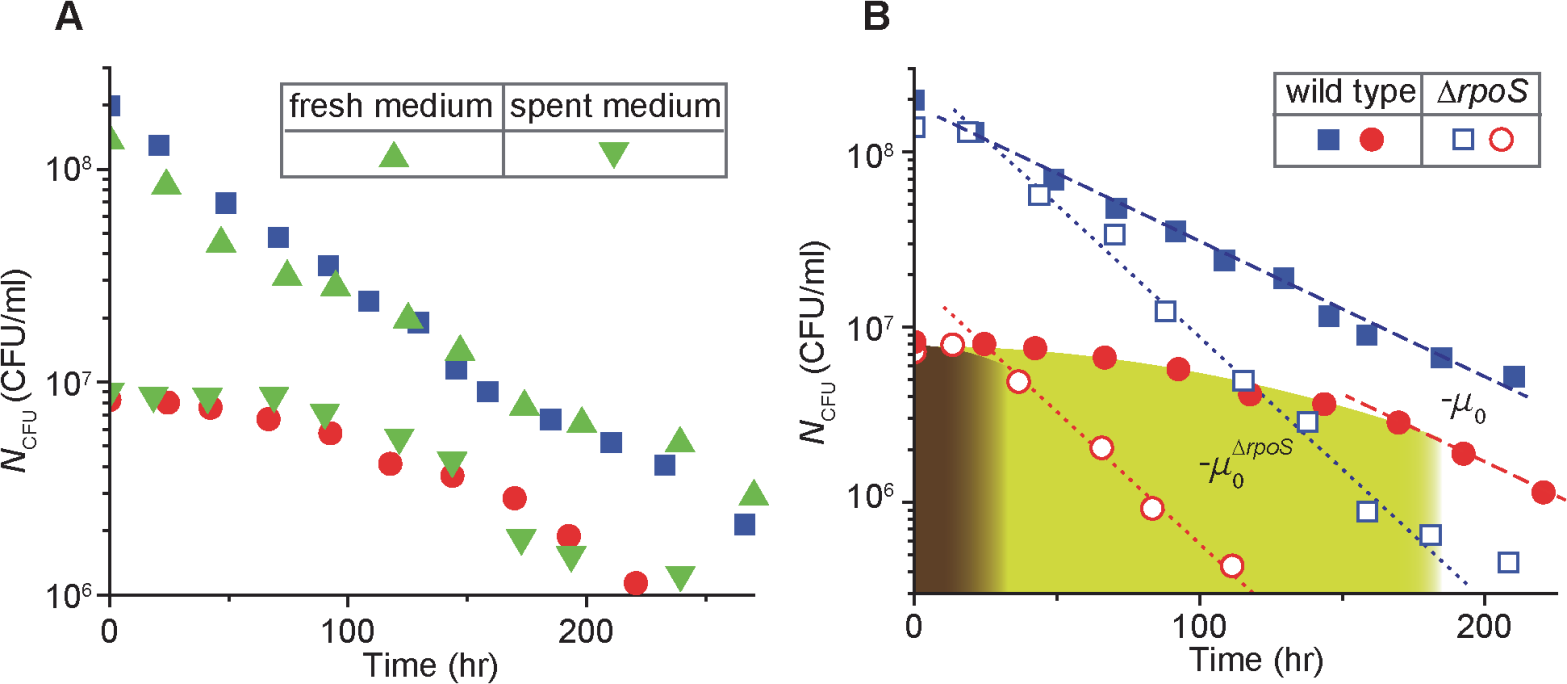
A role of extracellular signaling and *rpoS* in the density-dependent survival kinetics. (A) A role of extracellular signaling: a high density of exponentially-growing cells (*N*_CFU_ ≈ 7·10^8^/ml) was transferred to a fresh medium without glycerol. *N*_CFU_ of cells in the fresh medium (green triangles) decreases similarly to that from the previous experiment (solid blue squares, re-plotted from Fig. 1). Next, a spent medium was prepared from a culture of a high density of cells. *N*_CFU_ of cells at low density in the spent medium (green inverse triangles) decreases similarly to that from the previous experiments (solid red circles, re-plotted from Fig. 1). The results indicate that extracellular signaling does not play a role for the density-dependent kinetics. See the text for details. (B) A role of *rpoS*: Under starvation, *N*_CFU_ of the Δ*rpoS* strain (open symbols) decreases faster than that of the wild type strain (solid symbols, re-plotted from Fig. 1); compare the slope of the dotted line −μ_0_^ΔrpoS^ (= −0.035 hr ^-1^) and the slope of the dashed line −*μ*_0_ (= −0.018 hr ^-1^). See also S6 Fig for *N*_CFU_ of other densities of the Δ*rpoS* strain. Importantly, in the low cell-density cultures (e.g., open red circles), the periods during which *N*_CFU_ is maintained are much shorter for the Δ*rpoS* strain (brown region) than for the wild type strain (green region); note that here the brown and green regions are approximately determined as regions where the survival kinetics does not follow exponentially decay. This indicates that *rpoS* plays an important role for the wild type strain to maintain *N*_CFU_ for extended periods of time in low density under starvation.

First, cells were grown as in the previous experiment. When the growth stopped due to glycerol exhaustion at a high density (*N*_CFU_ ≈ 7·10^8^/ml), we washed the cells and re-suspended them in a fresh medium without glycerol; the fresh medium would not contain these secreted factors. In Fig. 2A, we see that *N*_CFU_ of cells in the fresh carbon-free medium (green triangles) decreases exponentially at a similar rate as *N*_CFU_ from the previous experiment (solid blue squares; re-plotted from Fig. 1), indicating that the lack of these secreted factors has little effect on the kinetics.

Second, we reason that a spent medium in which cells were grown previously to a high density would contain high levels of the secreted signals. Because the signals should be stable (discussed above), starving cells at low cell density in such a medium would exhibit a rapid, exponential decay of *N*_CFU_, similarly to that in high cell density. We prepared such a spent medium (the old cells were removed from the medium), added a low amount of glycerol and a low number of exponentially-growing cells to the medium, and grew them. After the growth was arrested at a low density (*N*_CFU_ ≈ 9·10^6^/ml) due to the exhaustion of glycerol, we measured *N*_CFU_ over time (see Materials and methods). We observed that *N*_CFU_ from the spent medium (green inverse triangles in Fig. 2A) follows the same biphasic pattern as *N*_CFU_ of the culture with a similar density from Fig. 1 (compare green inverse triangles and solid red circles; the solid red circles are re-plotted from Fig. 1). Taken together, these results indicate that the density-dependent kinetics of cell survival is not due to extracellular signaling.

### RpoS plays an important role in the maintenance of *N*_CFU_ observed in the first phase of the biphasic decay

Previously, it was known that the master regulator of the general stress response *rpoS* plays an important role for survival of *E*. *coli* cells under various environmental stresses [7–10]. To examine a role of *rpoS* in the observed kinetics, we repeated our experiment (that yielded Fig. 1) using the Δ*rpoS* strain and plotted *N*_CFU_ as open symbols in Fig. 2B and S6 Fig. For all the densities tested, *N*_CFU_ of the Δ*rpoS* strain decreases exponentially at the rate of −*μ*_o_^Δ*rpoS*^ (= −0.035 hr ^-1^; see the dotted lines). This is higher than that of the wild type strain, −*μ*_o_ (= −0.018 hr ^-1^), consistent with previous observation [18,25].

**Fig 3:**
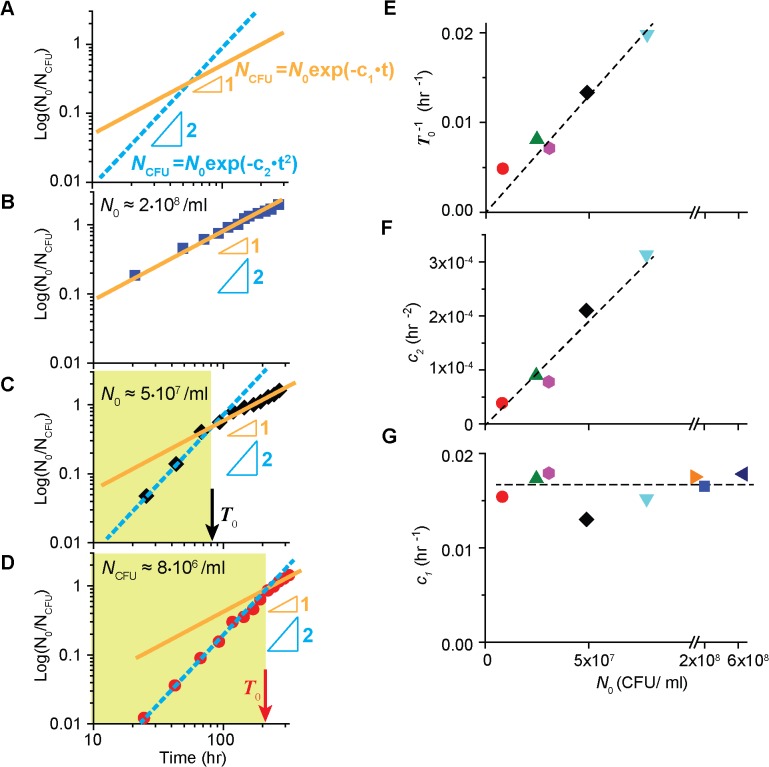
Quantitative analyses of the survival kinetics of wild type cells. (A) The log-log plot of log (*N*_0_ */N*_CFU_), where *N*_0_ is the number of CFU at the time zero, reveals the power law exponent of exponential functions; see the text, and Eqs (1) and (2). (B) At high cell density, *N*_CFU_ of the wild type cells plotted as described above follows a straight line with a slope of 1, indicating that the survival kinetics can be described by Eq. (1); see also S7A Fig for another high cell density. (C-D) At lower cell density (see also S7C Fig—S7E Fig for other low cell densities), the slope is initially 2 (green region), but becomes 1 later, revealing the biphasic decay seen in Fig. 1. The time at which the transition occurs is marked as *T*_*0*_ (arrows). Numerically, *T*_*0*_ is obtained from the time point at which the orange line and the cyan dashed line intersect. Note that *T*_*0*_ is greater for lower density. (E) T_0_^−1^ is linearly proportional to *N*_0_. (F, G) We obtained the coefficients *c*_1_ and *c*_2_ in Eqs (1) and (2) by fitting the second phase and the first phase of the biphasic decay respectively. Note that for high cell densities which show a single-phase decay, we used Eq. (1) to fit the entire range, and *c*_2_ is not available. We see that *c*_2_ increases linearly to *N*_0_ (*c*_2_ ∝ *N*_0_) in Fig. 3F. In Fig. 3G, we see *c*_1_ remains constant for different *N*_0_. The dashed lines are plotted for a visual guide.

*N*_CFU_ of the low cell density culture of the Δ*rpoS* strain (open red circles in Fig. 2B) exhibits a period of gradual decay before it decreases exponentially at the rate of −*μ*_o_^Δ*rpoS*^ (brown region). However, the period is much shorter than the period of gradual decay for the wild type cells (green region); note that the exact determination of this period is discussed below and in Fig. 3. This indicates that *rpoS* plays an important role for the wild type strain to maintain *N*_CFU_ for extended periods of time in low cell density.

### Quantitative analyses reveal simple empirical formulas describing the kinetics

To quantitatively analyze the survival kinetics of the wild type cells under starvation, we re-plotted the data in a manner that reveals the power law exponent of exponential functions; we denoted the number of colony-forming units at the time zero by *N*_0_ and plotted log (*N*_0_ /*N*_CFU_) against time in a log-log plot (Fig. 3 and S7 Fig). For example, if the kinetics of survival follows a first-order kinetics, meaning

$$N_{\text{CFU}}\propto \exp(-c_{1}\cdot t),$$ (1)

where *c*_1_ is a coefficient, the plot of log (*N*_0_ /*N*_CFU_) yields a straight line with a slope of 1 (orange line in Fig. 3A). If the kinetics follows

$$N_{\text{CFU}}\propto \exp(-c_{2}\cdot t^{2}),$$ (2)

where *c*_2_ is a coefficient, the plot yields a straight line with a slope of 2 (cyan dashed line).

For high cell-density cultures (i.e., *N*_0_ ≥ ∼10^8^ cells/ml), the data follows a straight line with a slope of 1 (Fig. 3B and S7A Fig), indicating the temporal kinetics of *N*_CFU_ is well described by Eq. (1), i.e., exponential decay, as discussed above. When we fit the data (in Fig. 1 and S2 Fig) using Eq. (1)_,_ we see *c*_1_ remains constant for different *N*_0_ (navy left triangle, blue square and orange right triangle in Fig. 3G) and *c*_1_ ≈ *μ*_o_ (= 0.018 hr ^-1^).

For lower cell-density cultures (i.e., *N*_0_ < ∼10^8^ cells/ml), the slope is initially 2 (green region in Fig. 3C and 3D, and S7C Fig—S7E Fig), but becomes 1 later, revealing the biphasic decay seen in Fig. 1 at low density. Thus, the first phase and second phase of the survival kinetics are well described by Eqs (2) and (1) respectively.

In these figures, the time at which the transition from the slope 2 and slope 1 occurs is marked as *T*_*0*_ (see arrows; it is the time point at which the two lines intersect). In Fig. 3E, we see T_0_^−1^ is linearly proportional to *N*_0_. Alternatively,

$$T_{0}\propto N_{0}^{-1},$$ (3)

indicating the period of the first phase becomes shorter as cell density increases. This suggests that we observe only the second phase of survival kinetics in high density (i.e., exponential decay in Fig. 1), because *T*_0_ is small.

To obtain coefficients in Eqs (1) and (2) for the low cell-density cultures, we fit the data in Fig. 1 and S2 Fig using the equations; the data in *t* < *T*_0_ and in *t* ≥ *T*_0_ are fitted using Eqs (2) and (1) respectively. We see that *c*_2_ increases linearly to *N*_0_ in Fig. 3F. Hence, *c*_2_ = *c* ⋅ *N*_0_, where *c* is a constant. Also, we see that *c*_1_ remains constant for different *N*_0_, and *c*_1_ ≈ *μ*_o_ in Fig. 3G.

Thus, together with Eq. (3), the temporal kinetics of *N*_CFU_ is well described by

$$N_{\text{CFU}}=\{\begin{array}{l} N_{0}\cdot \exp\left(-c\cdot N_{0}\cdot t^{2}\right)\\ N_{1}\cdot \exp\left(-\mu_{0}\cdot t\right) \end{array}\begin{array}{l} \;\text{if}\;0\leq t<T_{0}\\ \;\text{if}\;t\geq T_{0} \end{array},$$ (4)

where *N*_1_ is set to make *N*_CFU_ a continuous function, being equal to $N_{0}\exp(-c\cdot N_{0}\cdot T_{0}^{2}+\mu_{0}\cdot T_{0})$.

### Prolonged survival of starving cells by a RpoS-mediated negative feedback loop

The quantitative formula (Eq. (4)) reveals that the previous assumption—*N*_CFU_ decreases exponentially under starvation—is valid only at high cell density. At low cell density, however, *N*_CFU_ gradually decreases initially, before it decreases exponentially. The initial gradual decrease, well described by exp(-*t* ^2^), is extended at lower density, resulting in prolonged survival of starving cells. What is the mechanistic basis of the prolonged survival that appears in the density-dependent manner? Because the kinetics is significantly altered in the Δ*rpoS* strain (Fig. 2B), we first considered known regulation of RpoS expression and its effects on cell survival.

**Fig 4:**
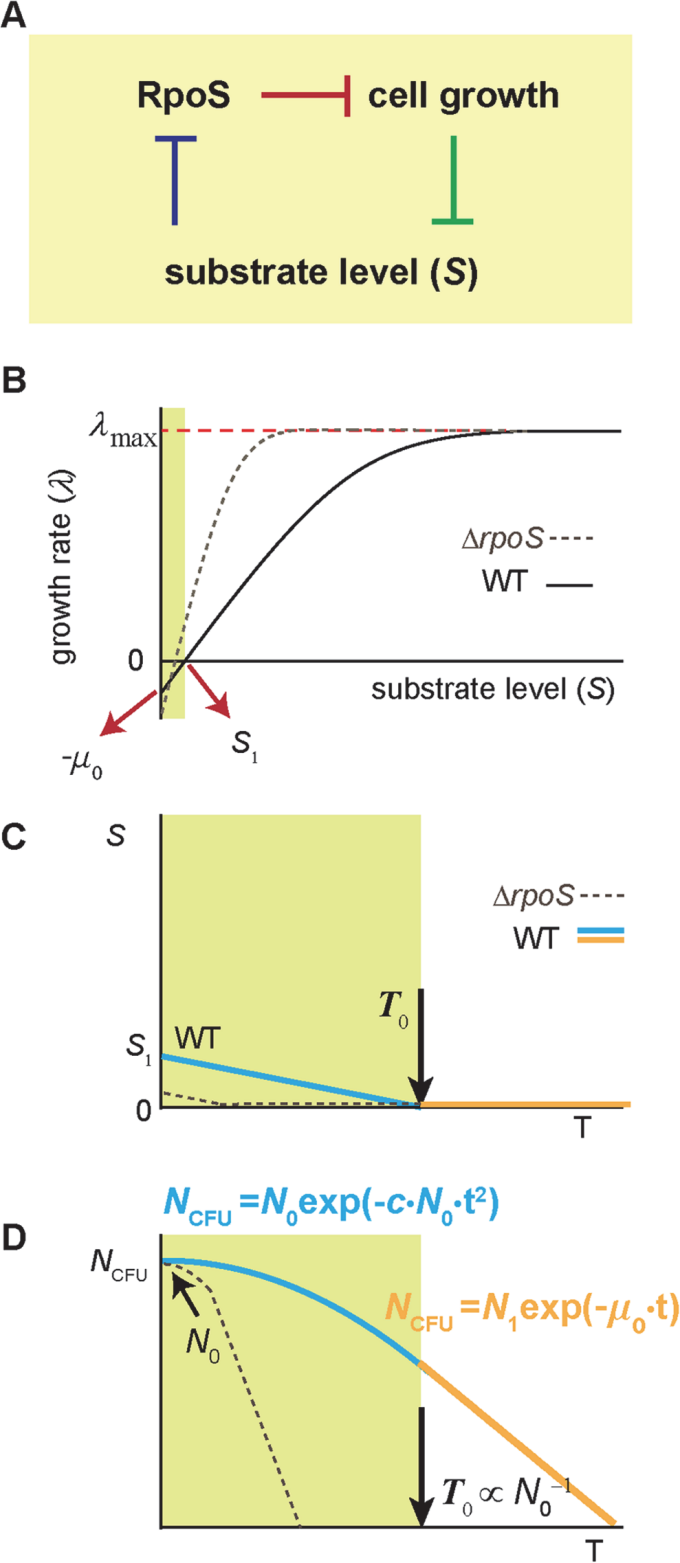
A mechanistic account of the density-dependent, biphasic survival kinetics. (A) Cells consume substrates for cell growth and the substrate concentration decreases in the medium (green line). When the concentration decreases to the levels affecting the rate of cell growth, RpoS accumulates (blue line) [26,27]. RpoS represses cell growth (red line) [30–32], forming negative feedback. In the feedback scheme, at low substrate levels, RpoS strongly represses cell growth and hence, substrate consumption, allowing cells to *conserve* a small amount of the substrate before it is completely depleted by cell growth. See the text for details. (B) This feedback predicts that as the substrate concentration is reduced, the growth arrest occurs at a non-zero substrate concentration *S*_1_, i.e., *λ* = 0 at *S* = *S*_1_ > 0. This prediction agrees with previous studies [33–35]. Importantly, further studies show that although the growth rate of the population is zero at *S* = *S*_1_, the substrate consumption rate is not zero; see [36] for review. This is commonly known as maintenance requirement; it requires continuous influx of the substrate to maintain a constant population size (*λ* = 0). If the influx of the substrate is less than the level needed for the maintenance, *λ* < 0 (green region) [37,38]. Our model indicates that *λ*(0) = − *μ*_0_; see the text for details. As a comparison, the relation of *λ* and *S* in the Δ*rpoS* strain is shown as a dashed line. Note that at intermediate substrate concentrations, *λ* of Δ*rpoS* strain is higher than that of the wild type strain [30–32]. Also, note that when the substrate is completely exhausted, the culture of the Δ*rpoS* strain loses viability more rapidly than the wild type strain (see [18,25] and Fig. 2B); thus, the value of *λ*(0) of Δ*rpoS* strain should be less than that of the wild type strain. (C, D) At the onset of growth arrest (time zero in S1B Fig), *S* = *S*_1_; see Fig. 4B. Without additional influx of the substrate, *S* will continue to decrease over time due to the consumption for the maintenance (cyan line in green region in Fig. 4C). Following the relation between *λ* and *S* depicted in Fig. 4B, *λ* will continue to decrease over time too. This will result in gradual decrease of *N*_CFU_ (cyan line in green region in Fig. 4D). At some point (*T*_0_), the substrate gets completely depleted (orange line in Fig. 4C) and *N*_CFU_ decreases exponentially at a fixed rate of *λ* (0) afterwards (orange line in Fig. 4D). For the culture with higher cell-densities, *S* will decrease faster because the substrate is consumed by more cells, leading to shorter periods of the first phase. Quantitative formulation of these processes straightforwardly leads to a mathematical solution equal to the empirical formulas (Eqs (3) and (4)). The solid lines in Fig. 1 and S2 Fig show the fits of the solution to the data. See the text for details.

As cells grow and consume substrates, the concentration of substrates in the medium will decrease (green line in Fig. 4A). When the concentration falls to the level reducing the rate of cell growth, the expression of RpoS is activated (blue line; note that higher RpoS levels at lower substrate concentrations were previously established [26,27]). The RpoS expression subsequently leads to expression of other new genes (i.e., RpoS regulon) and the expression of these genes protects cells from stress [7–10]. Importantly, this protection is expected to be density-independent, because RpoS expression itself is independent of cell density [26,27]. In Fig. 3G, we see that in the second phase of the survival kinetics, *N*_CFU_ decreases at the rate of −*μ*_o_ (= −0.018 hr ^-1^, dashed line) independently of cell density. This is lower than the rate of decrease in the Δ*rpoS* strain, −*μ*_o_^Δ*rpoS*^ (= −0.035 hr ^-1^, see Fig. 2B), suggesting that the protection lowers the rate of viability loss during the second phase independently of cell density. This protection, however, is not likely to be a major cause for the extension of the first phase at low density, because the extension is strongly dependent on cell density; see Fig. 1 and Fig. 3E. (There are studies suggesting that RpoS expression may be possibly higher at higher cell density [28,29]. Even if this is true, it cannot account for our observation that the first phase is extended further at lower cell density.)

Next, we turn to another major effect of RpoS. It is well known that the expression of RpoS represses cell growth (red line in Fig. 4A) [30–32]. Currently, the molecular mechanism of the repression is not clear, although it was proposed that RpoS directly inhibits the uptake of nutrients [39]. Importantly, with this repression, a negative feedback loop among RpoS, substrate concentration and cell growth is formed as depicted in Fig. 4A (blue and green lines were described above). In biological systems, negative feedback is frequently employed to achieve a homeostatic maintenance or a gradual change of a system, e.g., see ref. [40,41]. The negative feedback loop suggested above may play a similar role, providing a mechanism for a gradual change of *N*_CFU_ observed in the first phase of the survival kinetics (Fig. 1) for the wild type cells in the following way.

For biomass increase, cells consume substrates in the medium. As the substrate concentration in the medium decreases to low levels due to the consumption (green line in Fig. 4A), the feedback loop would exert repression on biomass increase (blue and red lines), and hence, the substrate consumption (green line). The repression would be stronger as the substrate concentration in the medium is further reduced. Eventually, it will lead to cessation or near-cessation of the substrate uptake for biomass increase, and consequently, prevents cells from completely depleting the substrate in the medium. Indeed, when we measured glycerol concentration in the medium at the onset of growth cessation (time zero in Fig. 1), we see that the glycerol concentration is not zero, but in μM range (see Materials and methods). This observation also agrees with previous studies [33–35]; in these studies, it is shown that as the substrate concentration *S* decreases, the growth rate *λ* decreases, but *λ* becomes zero at a *non-zero* substrate concentration. Denoting this concentration by *S*_1_, this phenomenon is illustrated in Fig. 4B as *λ* = 0 at *S* = *S*_1_ > 0.

Note that this is contrary to a prediction from the Monod equation, a well-known kinetic equation describing the relation between *λ* and *S* [42], which predicts *λ* = 0 when *S* = 0; see S2 equation. However, the Monod equation does not address the decrease in a population size during starvation (because *λ* in the Monod equation is always greater than or equal to 0), and is not applicable to our study. In fact, the Monod equation is a purely empirical formula based on curve fitting of experimental data (see the description below S2 Equation). A great deal of studies show that the Monod equation does not describe the dynamics of change in a population size well at very low substrate concentrations; see ref. [36,43] for review and S1 Text for details.

Importantly, even when the growth rate of a population is zero (i.e., *λ* = 0 at *S* = *S*_1_ in Fig. 4B), the substrate consumption rate is not zero [37,38,44,45]; also, see ref. [36] for review. These studies have shown that it requires continuous influx of the substrate into the medium to maintain the population size at a constant level, termed *maintenance requirement*. (It was proposed that the substrate is used to fix chemical “wear and tear” of cell materials and fulfill other non-growth related functions. See [36,46] for detail.). If the influx rate of the substrate meets the maintenance requirement, the population size is maintained; in Fig. 4B, this occurs when *S* is kept at *S*_1_ by continuous influx of the substrate against the consumption of the substrate for the maintenance. If the influx rate is less than the level needed for the maintenance, the population size decreases (*λ* < 0, green region in Fig. 4B). The rate of the decrease is faster at a lower influx rate of the substrate and, with no influx, the rate of the decrease reaches its maximum, i.e., *λ* (0) in Fig. 4B.

In our experiments, after the onset of growth arrest (time zero in S1B Fig), there is no additional influx of the substrate to the medium. But, as discussed above (3 paragraphs above), a certain amount of the substrate (i.e., *S*_1_) remains in the medium at the onset of growth arrest. This conserved substrate can be used for the maintenance, allowing cells to maintain their population size initially. Such usage will result in continuous decrease of *S* (cyan line in Fig. 4C), leading to a gradual decrease of *λ* below 0 (see *λ* < 0 when *S* < *S*_1_ in Fig. 4B). Consequently, the population size will gradually decrease, giving rise to the first phase of the biphasic decay (cyan line in Fig. 4D). Eventually, the substrate is completely exhausted at *T*_0_ (Fig. 4C), hence, *S* = 0 (orange line in Fig. 4C). Thus, after *T*_0_, *N*_CFU_ will decrease at the constant rate of *λ* (0), i.e., exponential decay, giving rise to the second phase (orange line in Fig. 4D).

In this model, the density-dependence of the first phase in the survival kinetics arises because, for the culture with higher cell densities, the conserved substrate (*S*_1_) will be consumed by more cells. Thus, it will be depleted faster for high cell density, leading to shorter periods of the first phase. Therefore, in our model, the density-dependent survival kinetics can be accounted for without invoking presence of (unknown) extracellular signaling molecules, agreeing with our observation in Fig. 2A. On a related note, there exists a study that shows a density-dependent response to bacterial survival under antibiotic treatment and such density dependence can be accounted for without invoking extracellular signaling [47].

### Mathematical modeling of the known processes accounts for the kinetics observed

To examine whether the biological processes described above can quantitatively account for the survival kinetics observed in our experiments, we constructed a mathematical model based on them. The details of our model are described in S1 text. Briefly, our model contains two key components, both of which are discussed above. The first component is the dependence of *λ* on *S*, which is plotted in Fig. 4B. Because we are particularly interested in the change of the population size when the substrate is nearly or completely exhausted, (i.e., *S* is close or equal to 0), the dependence of *λ* on *S* can be approximated to the first order in our model (see S3 Equation—S5 Equation). The second component of our model is the decrease of the substrate concentration due to the consumption for the maintenance (Fig. 4C). Here, based on previous studies [37,38], we assume the substrate consumption rate per cell is constant over time and the total consumption rate is proportional to cell density (S6 Equation).

Quantitative formulation of these processes straightforwardly leads to a mathematical solution equal to the empirical formulas; compare Eqs (3) and (4), and S11 Equation and S12 Equation. The solution states that a) the decay of *N*_CFU_ is biphasic, exp(−*c* ⋅ *N*_0_ ⋅ *t* ^2^) decay followed by exp(−*μ*⋅*t*) decay, and b) the time at which the transition occurs (i.e., *T*_0_) is inversely proportional to cell density. The solution contains two fitting parameters, *μ* and *c*. Representing the rate of a population decrease at the zero substrate concentration (i.e., *λ* (0); see the description below S4 Equation), the *μ* can be obtained from the rate of decrease of *N*_CFU_ in the second phase of survival kinetics in Fig. 1 and S2 Fig. Hence, *λ*(0) = − *μ* ≈ − *μ*_0_. Alternatively, the *μ* as well as *c* can be obtained by fitting the solution (S11 Equation and S12 Equation) to the data shown in Fig. 1 and S2 Fig. The result of the fit is plotted as lines in these figures, which yielded *μ = μ*_o_ = 0.018 hr ^-1^ and *c* = 4.7×10^–12^ ml·hr^-2^ (*λ*(0) = −*μ* = −*μ*_0_ in Fig. 4B is based on this result). The fit shows that the model can quantitatively account for our data—such consistency is expected because the solution of our model is equal to the empirical formula.

### Conclusion

The lifecycle of bacteria consists of short periods of feast, intercepted by long periods of starvation [1]. Quantitative analysis of how cells persevere during starvation is the focus of this study. Our findings show that after the onset of starvation, in high density cultures the loss of viability begins immediately at a constant rate. However, in low density cultures, the viability is maintained for extended periods of time before it decreases at the same constant rate. Such density-dependent survival kinetics is mediated by the master regulator of the general stress response *rpoS*. Integration of previously known processes reveals a thrifty strategy of bacteria, by which upon sensing impending starvation, cells repress nutrient consumption for biomass increase and use the remaining nutrient in the environment to delay cell death. Mathematical modeling of these processes accurately accounts for the density-dependent, biphasic survival kinetics.

The benefit of such thrifty behavior is obvious; it delays cell death. However, we note that such behavior has a cost in bacterial fitness because it diverts the limited nutrients away from cell growth, reducing the number of offspring. Thus, we expect the evolution of such behavior would depend on environmental conditions and be favored when the benefit outweighs the cost. The benefit is expected to depend on the length of starvation that cells routinely experience. If starvation periods are very *short*, the benefit of delaying death for *long*-term survival becomes negligible and may be outweighed by the cost. (in such case, it is expected that *rpoS* mutants outcompete the wild type cells.) The benefit would increase as the starvation periods become longer. Of course, if starvation periods are very long, it would lead to the emergence of GASP mutants [20,21], which is outside of the scope of this study. Thus, we expect that the evolution of such behavior would be strongly dependent on the starvation periods cells routinely experience. Another factor to affect the evolution of such behavior would be spatial structure of the environments. In structured environments where cells grow clonally, such behavior would be beneficial. However, in homogenous environments where the nutrients conserved to delay cell death by one species could be accessed by other species of bacteria, such behavior would not be beneficial. In such case, it would be more advantageous to use up all the nutrients and the *rpoS* mutants may be more fit than the wild type cells. Obviously, conserving the limiting nutrients by the wild type cells is one form of cooperation and *rpoS* mutants may appear as cheaters. However, the mixed population of the wild type cells and *rpoS* mutants may get fragmented and disperse at some point, and new monoclonal populations of wild types cells and those of *rpoS* mutants will be formed. During starvation, the latter will die rapidly, while the former will survive longer (Fig. 2B). As such, how often such population fragmentation occurs will affect the evolution; see the previous studies [48,49] that quantitatively examined how such fragmentation affects the evolution of cooperative behavior. These considerations, taken together, suggest that the evolution of the thrifty strategy observed in our study depends on various environmental factors, and it would be interesting, in future studies, to determine the dependence quantitatively. It is worth noting that in previous experiments, when cells were grown in the nutrient-limited chemostat where nutrient levels were artificially kept very low, *rpoS* mutants were frequently found [50,51]. The observation agrees with our argument in that at low nutrient levels, wild type would try to conserve the nutrients by not growing, while *rpoS* mutants will continue to grow.

On a related note, we believe such studies would draw an interesting analogy with a recent work that characterized the conditions affecting evolution of spore-formation in spore-forming bacteria [52]. Some bacterial species, such as *B*. *subtilis*, form spores upon sensing nutrient limitation [53]. Spores are very resistant to stress, persisting through starvation for long periods of time. In the recent work [52], it was shown that it is beneficial to initiate spore-formation before nutrients are completely depleted by biomass increase and, in some conditions, extracellular signaling may evolve to assist this process. The first finding is analogous to our findings in that upon sensing impending starvation, these cells take action for long-term survival *before the nutrients are completely depleted*. Also, the second finding has bearing on understanding why in the cells we studied (*E*. *coli*), extracellular signaling was not evolved (Fig. 2A).

We believe our study will advance our understanding of starved bacteria, especially their starvation survival physiology. Because ecosystems are dominated by starving microbes [1], our findings will facilitate deeper understanding of microbial population dynamics in microbial ecology and environmental sciences. We expect that such knowledge will have important implications in public health sectors [54]; e.g., accurate prediction of how pathogens persevere in freshwater will lead to better understanding of how infectious diseases spread and developing better public health policies.

## Materials and Methods

### Strain, media, and growth condition

*Escherichia coli* wild-type K12 strain NCM3722 [55,56] was used in our experiment. To make the Δ*rpoS* strain, we purchased the deletion allele of Δ*rpoS* from Keio deletion collection [57], transferred it to NCM3722 using P1 transduction [58].

N^-^C^-^ minimal media [59], supplemented with 20mM of NH_4_Cl and various concentrations of glycerol, were used for cell growth. The glycerol concentrations used were 5 mM, 1 mM, 0.7 mM, 0.5 mM, 0.3 mM, 0.2 mM, 0.15 mM, and 0.05 mM. Note that although glucose is a common carbon source for cell growth, we did not use glucose in our experiments because of bacterial Crabtree effect [60]; cells growing on glucose excrete acetate, and the excreted acetate is used as the cell density increases and the glucose level decreases. This would complicate our study to characterize the cell density dependence of *N*_CFU_ decay.

Cells were grown at 37°C with shaking at 250 r.p.m. in a water bath (New Brunkswick Scientific). To monitor their growth, optical density (OD_600_) was measured using a Genesys20 spectrophotometer (Thermo-Fisher). When the OD_600_ values were too low for the measurement using a standard sample holder (16.100-Q-10/Z8.5, Starna Cells Inc), a sample holder (18B-SOG-40, Starna Cells Inc) that is 4 times longer (OD_600_×4) was used. Cells were first grown in LB broth for 4∼5 hrs (seed culture), transferred to a N^-^C^-^ minimal medium with 20 mM of glycerol and 20 mM of NH_4_Cl and grown overnight (pre-culture). The next morning, the cells growing exponentially in the pre-culture were transferred to the media specified above (experimental culture). The initial density of cells in the experimental culture was adjusted such that cells continued to grow exponentially for at least 4 more doublings in the experimental culture, before their growth stopped due to the depletion of glycerol (S1 Fig).

The experiment in which the effects of extracellular signals were tested (Fig. 2A) was performed in the following way. First, cells were grown in the minimal medium with 5 mM of glycerol. When their growth stopped at high density due to glycerol depletion (*N*_CFU_ ≈ 7·10^8^/ml), we waited ∼7 hrs. Then, cells were spun down (the supernatant was set aside), transferred to a carbon-free medium with 20 mM of NH_4_Cl. The volume of the carbon-free medium was adjusted in such a way that the initial cell density (measured from OD_600_) matches that from the viability curve of blue squares in Fig. 1. Then, their viability was measured afterwards (green triangles in Fig. 2A). Then, to the supernatant obtained from the procedure above, we added 0.05 mM of glycerol, transferred exponentially growing cells into it (*N*_CFU_ ≈ 5·10^5^/ml), and grew them until growth stopped due to glycerol depletion at low cell density (*N*_CFU_ ≈ 9·10^6^/ml). Then, we measured their viability afterwards (green inverse triangles in Fig. 2A). Please note that initial cell density was first estimated from the OD_600_ value of the culture (with the knowledge that 1 OD_600_ corresponds to ∼10^9^ cells/ml) and later confirmed (using the viability assay as described below).

### Viability measurement

The viability was determined by counting the number of colony-forming-unit (*N*_CFU_) on LB agar plates. After platting, the plates were incubated at 37°C overnight before counting. *N*_CFU_ did not change even if the plates were incubated for 3∼5 more days. Through serial dilutions, we ensured *N*_CFU_ to be around 100∼200 per agar plate (100 × 15 mm petri dish). *N*_CFU_ reported was averaged values of 3 replicate measurements. Each experiment was independently repeated 2∼4 times (e.g., see S8 Fig).

### Glycerol concentration measurement

Glycerol concentration in the medium was measured using Glycerol assay kit (SigmaAldrich, F6428) as described in the manual, except the ratio between the medium and the agent was increased to 1 to 1.5. In four independently repeated experiments, we observed that the glycerol concentration at the onset of growth arrest of glycerol-starved cultures was between 0.5∼2 μM. We note that this is below the range of quantitative measurement of the method employed, and absolute quantification of such low concentrations is very difficult. However, we always observed positive values. (The measurement was calibrated using media with known glycerol concentrations. In this calibration, the medium without glycerol is used as the reference for zero glycerol concentration.)

## Supporting Information

S1 Text Supporting text for formulation of our mathematical model. (DOCX)

S1 Fig Cell growth using glycerol as the sole carbon source. In our experiments, we grew cells in batch culture with glycerol as the sole carbon source. As cells grow, glycerol was consumed and eventually exhausted. (A) The cultures contained different amounts of glycerol initally, which resulted in different saturating cell densities at the onset of growth arrest; see a linear relation between the saturating cell density and the initial glycerol concentration in the medium. In our experiments, we always used low enough amounts of glycerol to ensure that the growth was arrested as a result of the exhaustion of glycerol. (B) We adjusted the inoculation density in our experimental culture such that cells grew exponenitally at least 4 doublings in the experimental culture before their growth stopped due to glycerol exhaustion. The transition from growth and nongrowth occurs abruptly. The onset of growth arrest defines the time zero in our experiments (red arrow). Note that 1 OD_600_ corresponds to ~10^9^ cells/ml.
(TIF)

S2 Fig The survival kinetics of starving *E*. *coli* cells. See the caption of Fig. 1 for details.
(TIF)

S3 Fig Cell-density dependent, biphasic decay pattern of *N*_CFU_ in acetate-depleted (solid circles) and maltose-depleted (open squares) cultures. The experiments were performed similarly as the experiments using glycerol as the sole carbon source (see Materials and methods), except that acetate or maltose was used as the sole carbon source. Briefly, cells were grown with different concentrations of acetate [12 mM (solid red circles), 1.2 mM (solid blue circles) and 0.12 mM (solid green circles)] or with different concentrations of maltose [1.25 mM (open red squares), 0.125 mM (open blue squares) and 0.0125 mM (open green squares)]. After their growth was arrested due to the exhaustion of the carbon sources, *N*_CFU_ was measured. The decay patterns of *N*_CFU_ are similar to that of the glycerol-depleted culture (Fig. 1; see the main text). In high cell density (red symbols), *N*_CFU_ follows a single-phase exponential decay. But in low density (green symbols), *N*_CFU_ follows a biphasic decay; *N*_CFU_ is maintained at near-constant levels initially and eventually decreases exponentially. This shows that cell-density dependent, biphasic decay patterns of *N*_CFU_ are not glycerol-specific, but occur for other carbon sources.
(TIF)

S4 Fig Comparing the survival kinetics of two *E*. *coli* strains. In the main text, we used wild-type K12 strain NCM3722 and characterize its survival kinetics (solid symbols). Here, we repeated the experiment using MG1655 (CGSC# 7740) (open symbols). We observe that the survival kinetics is similar for the two strains.
(TIF)

S5 Fig The survival kinetics of *E*. *coli* cells taken from the cultures starved of glycerol for ~12 days. Our starvation experiment usually lasted for 12 days. From the agar plates used for colony-counting at the last day of the experiment, we randomly picked 20 single colonies, re-cultured them and repeated the starvation experiment. We observed that *N*_CFU_ of these cells decreases similarly to *N*_CFU_ shown in Fig. 1; *N*_CFU_ of the cells from the 4 colonies is plotted as empty symbols here. For comparison, *N*_CFU_ from Fig. 1 is re-plotted (solid symbols).
(TIF)

S6 Fig Comparison of *N*_CFU_ of the wild type strain and the Δ*rpoS* strain at different densities. (A-C) *N*_CFU_ of the wild type strain (solid symbols) and the Δ*rpoS* strain (open symbols) at different densities are plotted. Dotted and dashed lines are overlaid for a guide. See the caption of Fig. 2 for details.
(TIF)

S7 Fig Quantitative analyses of the survival kinetics. (A-E) The log-log plots of log (*N*_0_ */N*_CFU_) at different densities. See the caption of Fig. 3 for details.
(TIF)

S8 Fig Checking the reproducibility of our data. To accurately determine the number of colony forming units, we used serial dilutions and ensured the number to be around 100~200 per agar plate. Also, we had three replicates and reported the average values from the 3 replicate measurements for *N*_CFU_ (see Materials and methods). Then, we repeated this procedure three times independently and plotted the data in the panel A. In the panel B, we analyzed the data similarly as described in the caption of Fig. 3. The analysis shows an agreement among all three independent experiments; at high cell density the slope is 1, and at low cell density the slope is initially 2. Thus, our data is highly reproducible.
(TIF)
