# Supplementary material for: Survival Kinetics of Starving Bacteria Is Biphasic and Density-Dependent
Source: PLoS Comput Biol. 2015 Apr 2;11(4):e1004198. doi: 10.1371/journal.pcbi.1004198 (PMC4383377; doi:10.1371/journal.pcbi.1004198)
Supplement: S1 Text — (DOCX) [file pcbi.1004198.s001.docx]

**S1 Text**

In the main text, we showed that our quantitative model based on known biological processes accounts for our experimental data. Below, we will describe the model in detail.

Monod equation

For a population reproducing at the specific growth rate of *λ_M_*, the number of viable cells in the population *N* can be described by

$\frac{dN}{dt}=\lambda_{M}\cdot N$. (S1)

Monod showed that there is a simple relationship between the specific growth rate and the concentration of a growth-limiting substrate, *S* [[42](#_ENREF_42)]. Known as the Monod equation, the relationship is

$\lambda_{M}\left(\frac{S}{K}\right)=\lambda_{\max}\frac{S/K}{1+S/K}$, (S2)

where *λ*_max_ is the maximum specific growth rate and *K* is a saturation constant. *K* is numerically equal to the substrate concentration at which the growth rate is equal to$\lambda_{\max}/2$. It is noteworthy that S2 Equation was proposed as a *purely empirical equation* based on curve fitting of experimental data. Monod noted in the paper, “Several mathematically different formulations could be made to fit the data. But it is both convenient and logical to adopt a hyperbolic equation” [[42](#_ENREF_42)].

Quantitative formulation of cell growth and death at low substrate concentrations

S2 Equation has been widely used to describe growth dynamics of microbial populations and shown to provide a satisfactory fit when the substrate concentration is relatively high (e.g., $\frac{S}{K}\geq1$) [[61](#_ENREF_61)]. However, a great deal of studies has shown that S2 Equation is not applicable at low substrate concentrations ($\frac{S}{K}<<1$), [[39](#_ENREF_39),[43](#_ENREF_43)]. Such discrepancy is expected because S2 Equation does not address the decrease of a population size due to cell death; $\lambda_{M}\left(\frac{S}{K}\right)$is always equal to or greater than zero. Thus, for our study, $\lambda_{M}\left(\frac{S}{K}\right)$should be modified to include cell death. We denote the modified form by $\lambda\left(\frac{S}{K}\right)$. Importantly, $\lambda\left(\frac{S}{K}\right)$should become negative as *S* → 0 to reflect the decrease in the population size under starvation (as illustrated in Fig. 4B).

Here, we wish to know how$\lambda$depends on *S*. Particularly, in our study focusing on quantitative description of the decrease in the population size *when the substrate is nearly or completely depleted*, it is important that we know such dependence at very low substrate concentrations, i.e., $\frac{S}{K}<<1$. Thus, below, we will derive the mathematical form of $\lambda\left(\frac{S}{K}\right)$for $\frac{S}{K}<<1$.

First, we write the Taylor series of the function $\lambda\left(\frac{S}{K}\right)$about $\frac{S}{K}=0$ as

$\lambda\left(\frac{S}{K}\right)=\lambda(0)+\lambda^{'}(0)\cdot\left(\frac{S}{K}\right)+\frac{1}{2!}\cdot\lambda^{''}(0)\cdot\left(\frac{S}{K}\right)^{2}+\frac{1}{3!}\cdot\lambda^{'''}(0)\cdot\left(\frac{S}{K}\right)^{3}\cdots$. (S3)

For $\frac{S}{K}<<1$, we can approximate S3 Equation to the first order,

$\lambda\left(\frac{S}{K}\right)=\lambda(0)+\lambda^{'}(0)\cdot\left(\frac{S}{K}\right)$. (S4)

We note that *λ*(0) < 0, as discussed above. Now, we denote *λ*(0) by −*μ*, where *μ* is a positive constant ($\mu\equiv-\lambda(0)$). Obviously, if the substrate concentration in the medium is increased from zero, $\lambda\left(\frac{S}{K}\right)$ will change from negative to positive at some point (because the population size will increase at high values of *S*). We define the substrate concentration at which the crossover (from negative to positive) occurs as *S*_1_. Existence of such non-zero substrate concentration agrees with previous studies [[36-38](#_ENREF_36)]; in these studies, it was shown that as the concentration of a growth-limiting substrate decreases, the growth rate decreases, and the growth rate becomes zero at a low, but positive substrate concentration.

Using *μ* and *S*_1_, we can rewrite the S4 Equation as

$\lambda\left(S\right)=\mu\left(\frac{S}{S_{1}}-1\right)$. (S5)

Constant substrate consumption at zero growth: Maintenance requirement

Further studies of cell growth at very low nutrient concentrations show that even when the net population growth is zero, the nutrient consumption rate is not zero; it requires a continuous influx of nutrients (and energy) to the medium to maintain a constant population size [[40](#_ENREF_40),[41](#_ENREF_41),[44](#_ENREF_44),[45](#_ENREF_45)]. It was proposed that the nutrients (and energy) is used to fulfill non-growth related functions such as maintaining membrane potential, osmotic regulations, motility, turnover of macromolecular components, etc. [[39](#_ENREF_39),[46](#_ENREF_46)].

Previously, the substrate consumption for the maintenance in glucose-starved *E. coli* cells was measured to be 0.005 g glucose / g of dry weight /hr [[41](#_ENREF_41)]. As a comparison, biomass yield of rapidly growing *E. coli* cells is 2 g glucose / g of dry weight [[62](#_ENREF_62),[63](#_ENREF_63)]. Thus, considering that *E. coli* cells grow at ~ 1 doubling / hr with glucose as a carbon source (and ammonium as a nitrogen source), for cells growing rapidly in substrate-rich conditions, the maintenance requirement is negligible compared to the demand for biomass increase. However, when the substrate concentration is very low, whether the maintenance requirement is met or not has significant effects on long-term survival of cells [[40](#_ENREF_40),[41](#_ENREF_41)].

In the main text, we described that at the onset of growth arrest, a small amount of the substrate was conserved and used for the maintenance requirement, leading to the initial maintenance of *N*_CFU_ at ~ *N*_0_ (green region in Fig. 2B). If we define the substrate consumption rate per cell for maintenance as *m*, in this region the substrate concentration changes by

$\frac{dS}{dt}=-m\cdot N_{0}$. (S6)

Note that in our experiment, the time zero was defined as the onset of growth arrest (i.e., *λ* = 0); see S1 Figure. Since *λ* = 0 at *S* = *S*_1_ in Eq. (S5), the substrate concentration at time zero is *S*_1_ (i.e., *S*(0) = *S*_1_). Thus,

$S=S_{1}-m\cdot N_{0}\cdot t$. (S7)

While it is possible that the viable cells may utilize lysis products from dead cells for maintenance, it was shown that *E. coli* cells do not take up the lysis products from dead cells during starvation [[19](#_ENREF_19)]. This is strongly supported by the constant death rate observed in high cell density (Fig. 1). Thus, the utilization of lysis products from dead cells by live cells is not included in S7 Equation.

Obviously, the substrate concentration cannot fall below zero, i.e., *S* ≥ 0. If we denote the time at which the substrate gets completely exhausted (i.e., *S* = 0) by *T*_0_, S7 Equation predicts

$T_{0}=\frac{S_{1}}{m\cdot N_{0}}$. (S8)

Thus,

$S=\left\{\begin{matrix} S_{1}-m\cdot N_{0}\cdot t & \text{if }0\leq t<T_{0} \\ 0\text{ } & \text{ if }t\geq T_{0}\text{ } \end{matrix}\right.$. (S9)

Cell-density dependent, biphasic kinetics of cell survival during starvation

Combination of S5 Equation and S9 Equation shows that the specific growth rate *λ*(*t*) can be described by

$\lambda(t)=\left\{\begin{matrix} -\left(\frac{\mu\cdot m\cdot N_{0}}{S_{1}}\right)\cdot t & \text{if }0\leq t<T_{0} \\ -\mu\text{ } & \text{ if }t\geq T_{0}\text{ } \end{matrix}\right.$ . (S10)

Putting together S1 Equation and S10 Equation, we have the following solution,

$N=\left\{\begin{matrix} N_{0}\cdot\exp\left(-c\cdot N_{0}\cdot t^{2}\right) \\ N_{1}\cdot\exp\left(-\mu\cdot t\right)\text{ } \end{matrix}\begin{matrix} & \text{if }0\leq t<T_{0} \\ \text{ } & \text{ if }t\geq T_{0}\text{ } \end{matrix}\right.$, (S11)

where $c\equiv\frac{\mu}{2}\cdot\frac{m}{S_{1}}$, and *N*_1_ is set to make *N* continuous, i.e., $N_{1}\equiv N_{0}\cdot\exp\left(\frac{\mu^{2}}{4}\cdot\frac{1}{cN_{0}}\right)$. Using the constant *c*, S8 Equation can be rewritten as

$T_{0}=\frac{\mu}{2c}\cdot\frac{1}{N_{0}}$. (S12)

The S11 Equation and S12 Equation contain two fitting parameters, *μ* and *c*, which can be obtained by fitting the equation to the data shown in Fig. 1 and S2 Figure (note that *N*_0_ is the number of CFU at the time zero and is measured experimentally. Thus, it is not a fitting parameter). The lines in these figures are the fit of S11 Equation and S12 Equation to the data, which yielded *μ* = *μ*_o_ = 0.018 hr ^-1^ and *c* = 4.7×10^-12^ ml⋅hr^-2^.

Importantly, *S11 Equation is equal to the empirical formulas derived from the experimental data in the main text (Eq. (4)).* Also S12 Equation accounts for the inversely proportional dependence of *T*_0_ on *N*_0_ observed in the experiment, i.e., Eq. (3) and Fig. 3E.

**Supporting references**

61. Gaudy A, Gaudy E (1980) Microbiology for environmental scientists and engineers: McGraw-Hill.

62. Shiloach J, Fass R (2005) Growing E. coli to high cell density--a historical perspective on method development. Biotechnol Adv 23: 345-357.

63. Luli G, Strohl W (1990) Comparison of growth, acetate production, and acetate inhibition of Escherichia coli strains in batch and fed-batch fermentations. Appl Environ Microbiol 56: 1004-1011.
