# Supplementary figures and images for: Survival Kinetics of Starving Bacteria Is Biphasic and Density-Dependent
Source: PLoS Comput Biol. 2015 Apr 2;11(4):e1004198. doi: 10.1371/journal.pcbi.1004198 (PMC4383377; doi:10.1371/journal.pcbi.1004198)

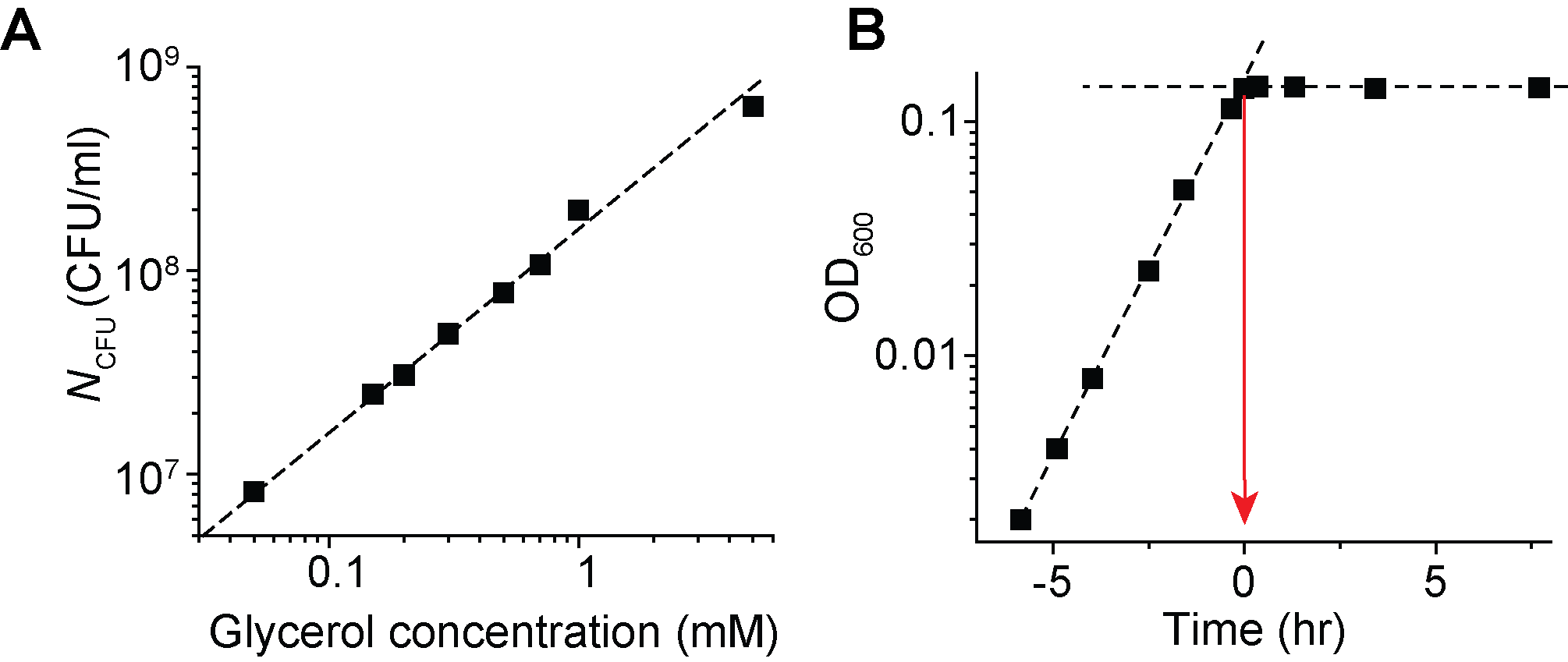

Supplement: S1 Fig — In our experiments, we grew cells in batch culture with glycerol as the sole carbon source. As cells grow, glycerol was consumed and eventually exhausted. (A) The cultures contained different amounts of glycerol initally, which resulted in different saturating cell densities at the onset of growth arrest; see a linear relation between the saturating cell density and the initial glycerol concentration in the medium. In our experiments, we always used low enough amounts of glycerol to ensure that the growth was arrested as a result of the exhaustion of glycerol. (B) We adjusted the inoculation density in our experimental culture such that cells grew exponenitally at least 4 doublings in the experimental culture before their growth stopped due to glycerol exhaustion. The transition from growth and nongrowth occurs abruptly. The onset of growth arrest defines the time zero in our experiments (red arrow). Note that 1 OD600 corresponds to ~109 cells/ml. (TIF) [file pcbi.1004198.s002.tif]

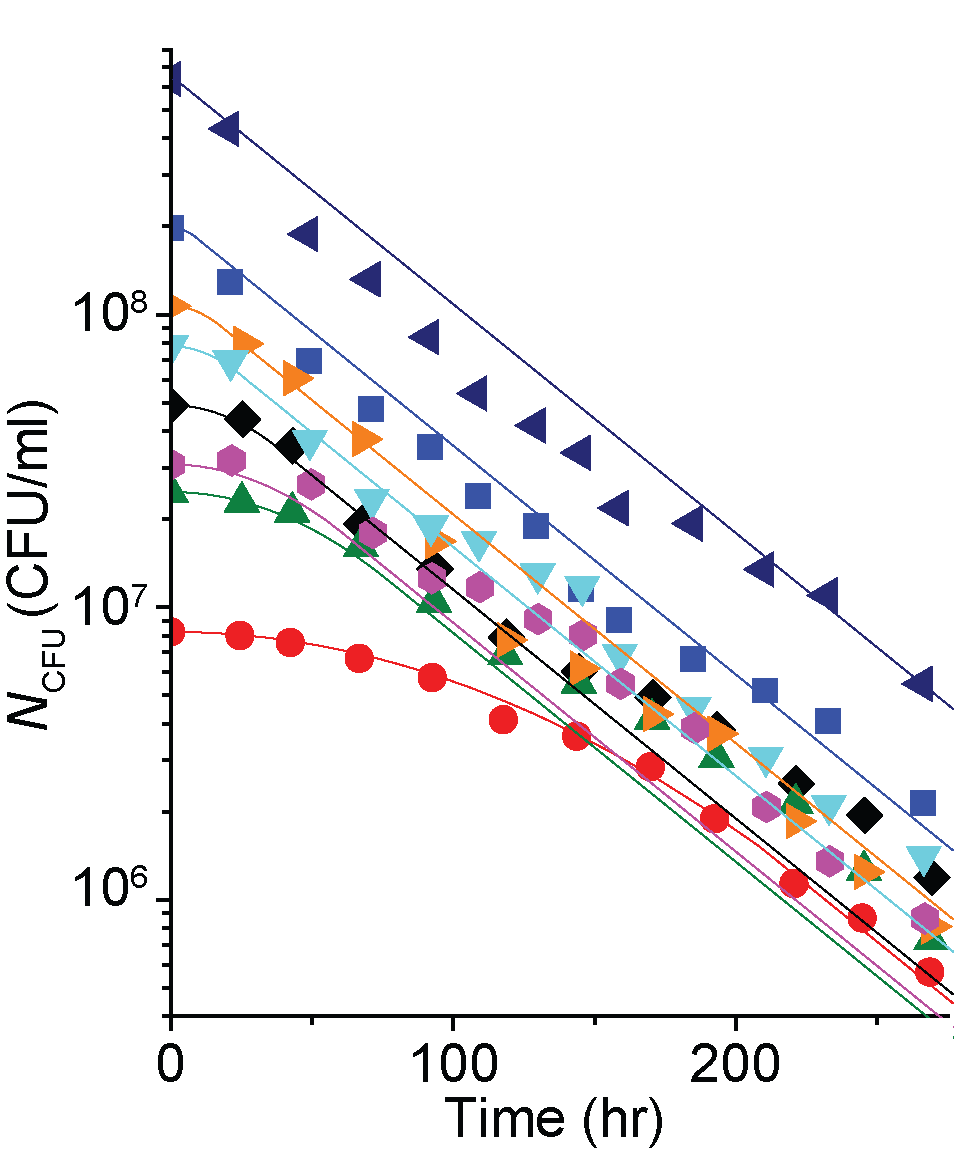

Supplement: S2 Fig — See the caption of Fig. 1 for details. (TIF) [file pcbi.1004198.s003.tif]

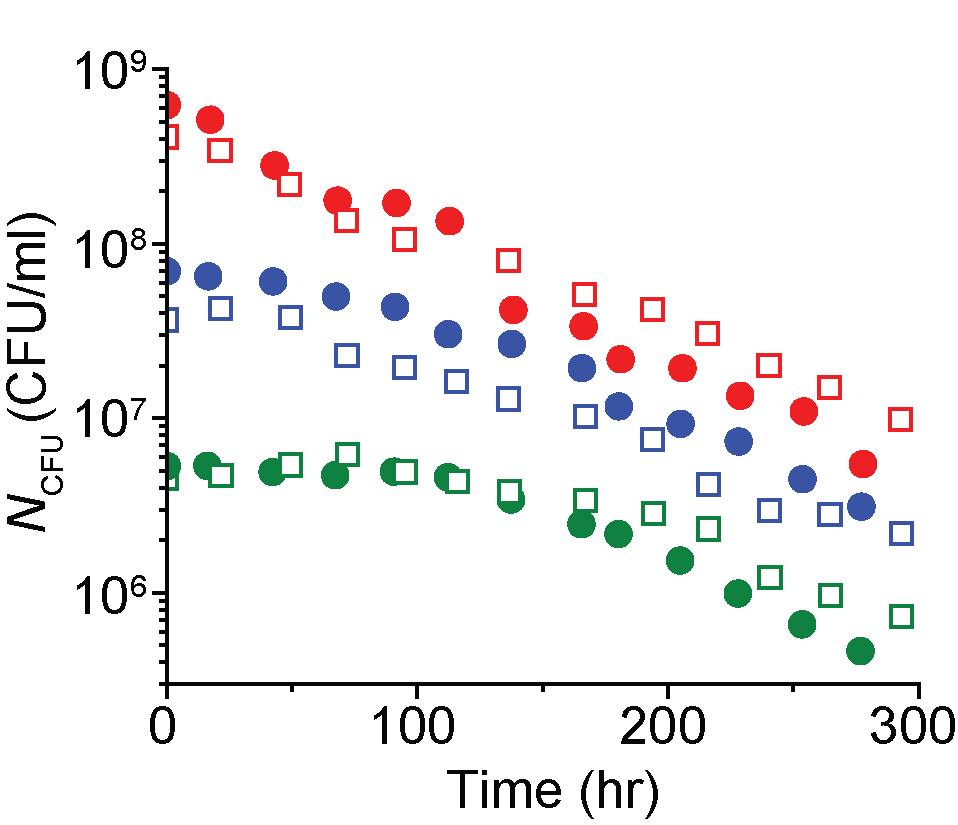

Supplement: S3 Fig — The experiments were performed similarly as the experiments using glycerol as the sole carbon source (see Materials and methods), except that acetate or maltose was used as the sole carbon source. Briefly, cells were grown with different concentrations of acetate [12 mM (solid red circles), 1.2 mM (solid blue circles) and 0.12 mM (solid green circles)] or with different concentrations of maltose [1.25 mM (open red squares), 0.125 mM (open blue squares) and 0.0125 mM (open green squares)]. After their growth was arrested due to the exhaustion of the carbon sources, N CFU was measured. The decay patterns of N CFU are similar to that of the glycerol-depleted culture (Fig. 1; see the main text). In high cell density (red symbols), N CFU follows a single-phase exponential decay. But in low density (green symbols), N CFU follows a biphasic decay; N CFU is maintained at near-constant levels initially and eventually decreases exponentially. This shows that cell-density dependent, biphasic decay patterns of N CFU are not glycerol-specific, but occur for other carbon sources. (TIF) [file pcbi.1004198.s004.tif]

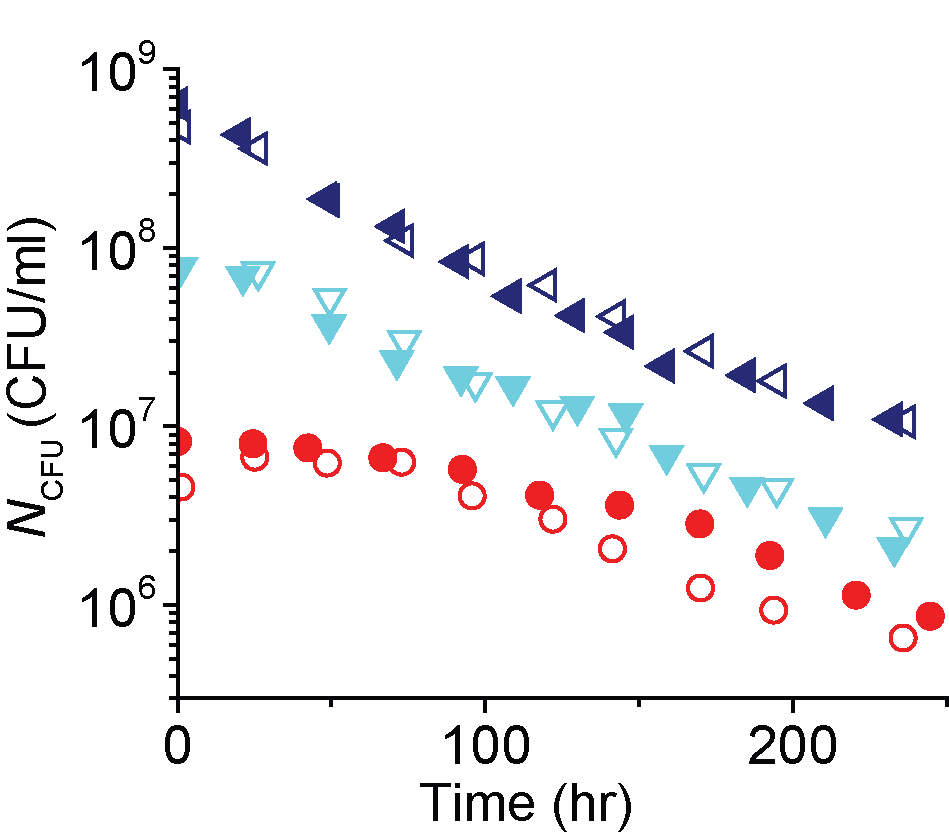

Supplement: S4 Fig — In the main text, we used wild-type K12 strain NCM3722 and characterize its survival kinetics (solid symbols). Here, we repeated the experiment using MG1655 (CGSC# 7740) (open symbols). We observe that the survival kinetics is similar for the two strains. (TIF) [file pcbi.1004198.s005.tif]

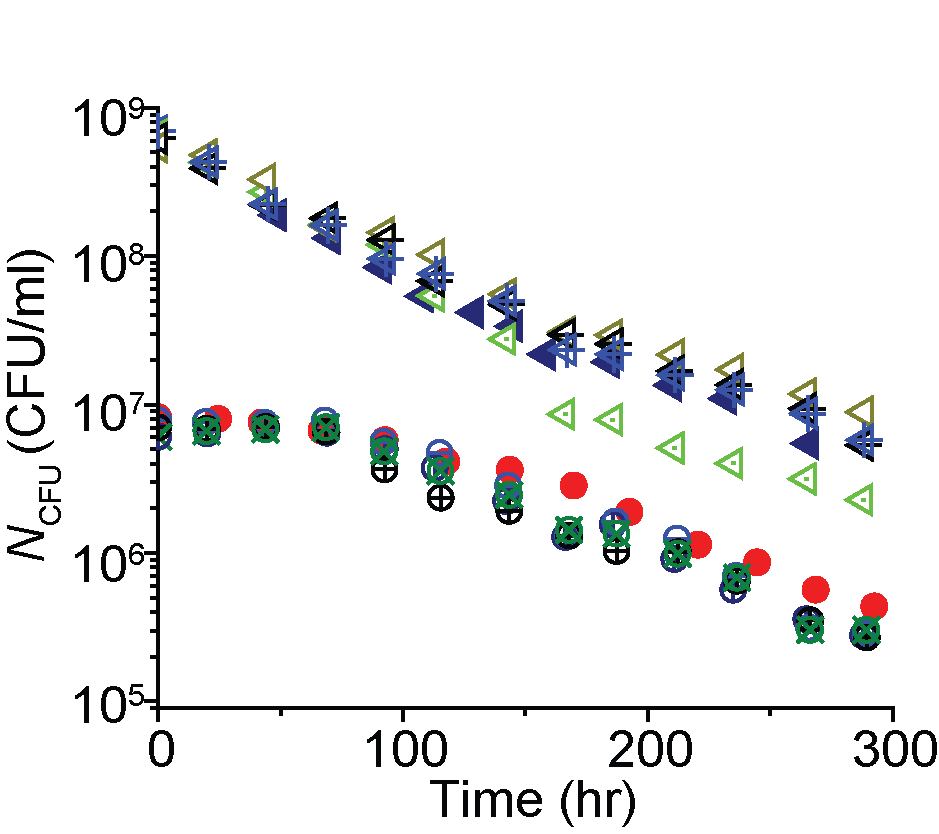

Supplement: S5 Fig — Our starvation experiment usually lasted for 12 days. From the agar plates used for colony-counting at the last day of the experiment, we randomly picked 20 single colonies, re-cultured them and repeated the starvation experiment. We observed that N CFU of these cells decreases similarly to N CFU shown in Fig. 1; N CFU of the cells from the 4 colonies is plotted as empty symbols here. For comparison, N CFU from Fig. 1 is re-plotted (solid symbols). (TIF) [file pcbi.1004198.s006.tif]

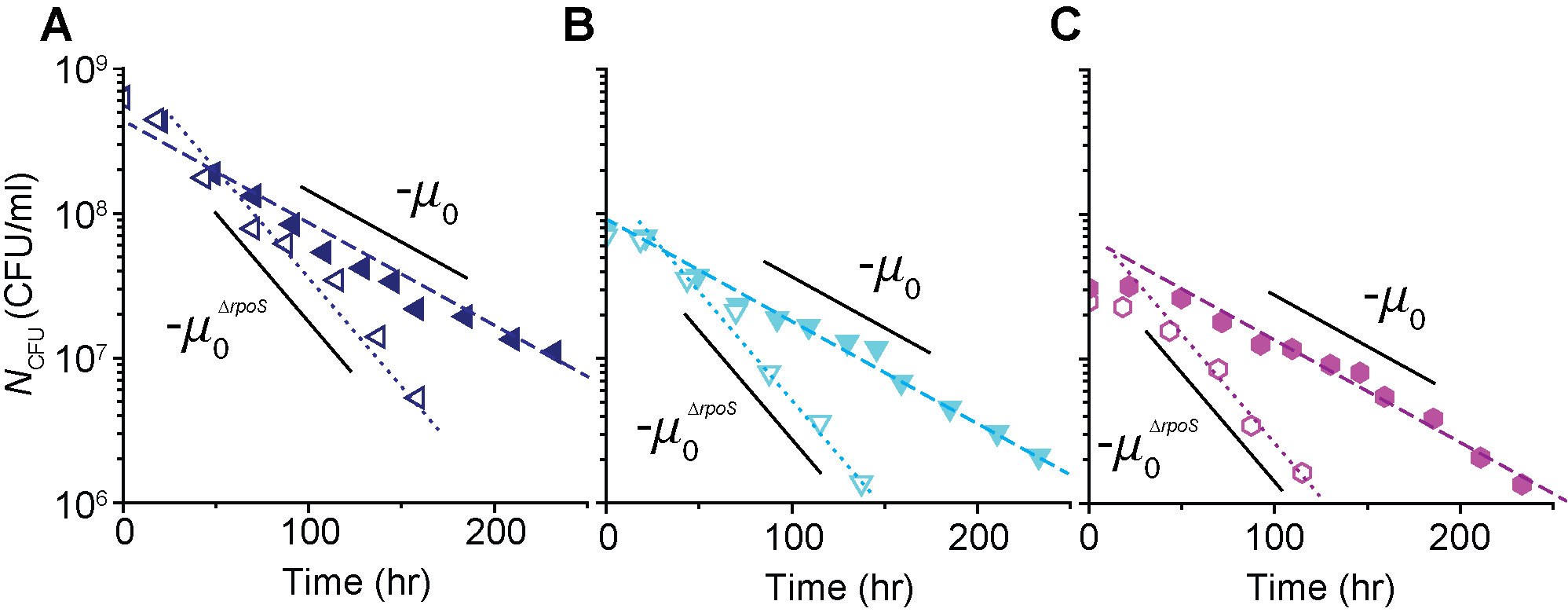

Supplement: S6 Fig — (A-C) N CFU of the wild type strain (solid symbols) and the ΔrpoS strain (open symbols) at different densities are plotted. Dotted and dashed lines are overlaid for a guide. See the caption of Fig. 2 for details. (TIF) [file pcbi.1004198.s007.tif]

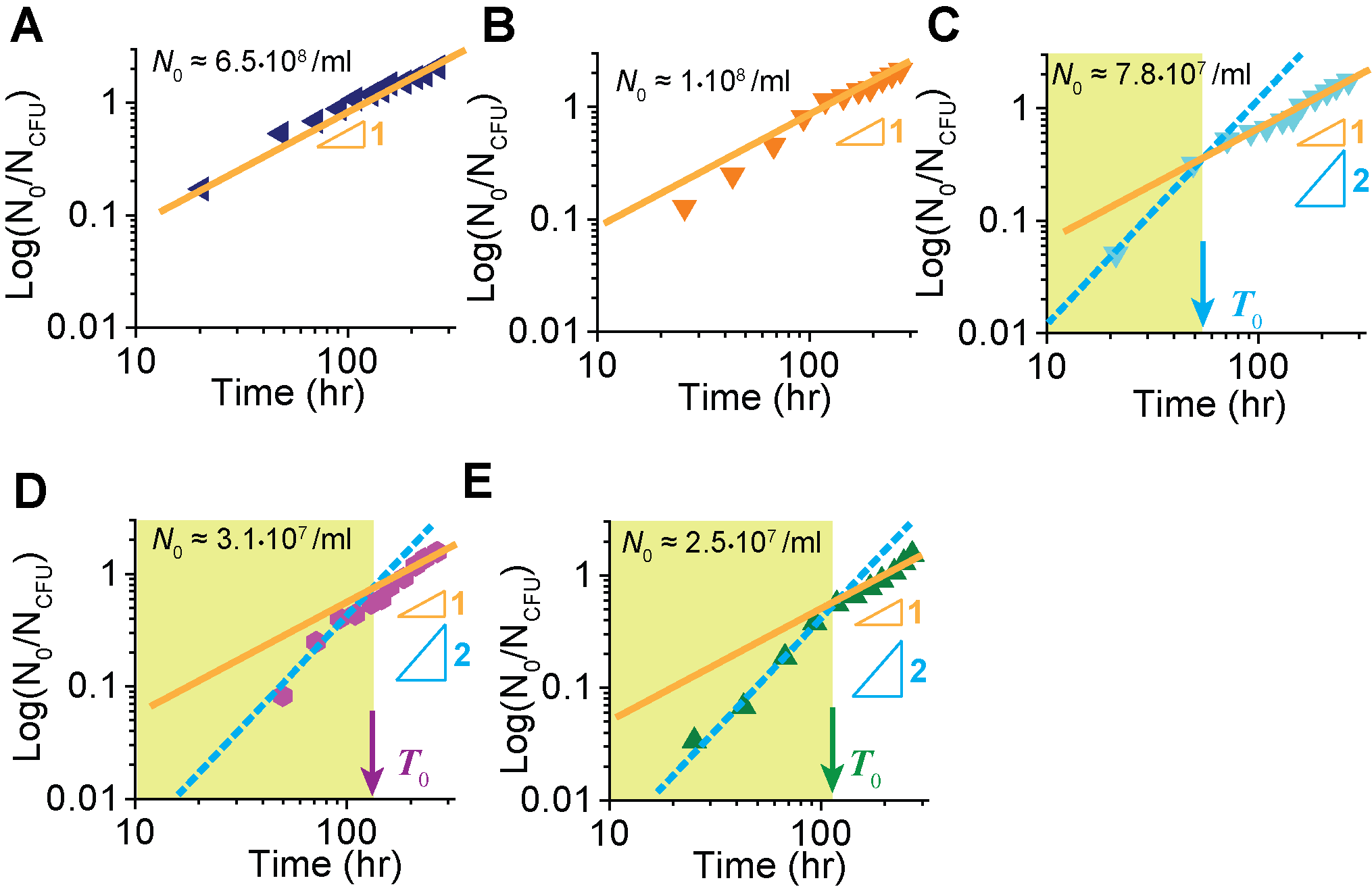

Supplement: S7 Fig — (A-E) The log-log plots of log (N 0 /N CFU) at different densities. See the caption of Fig. 3 for details. (TIF) [file pcbi.1004198.s008.tif]

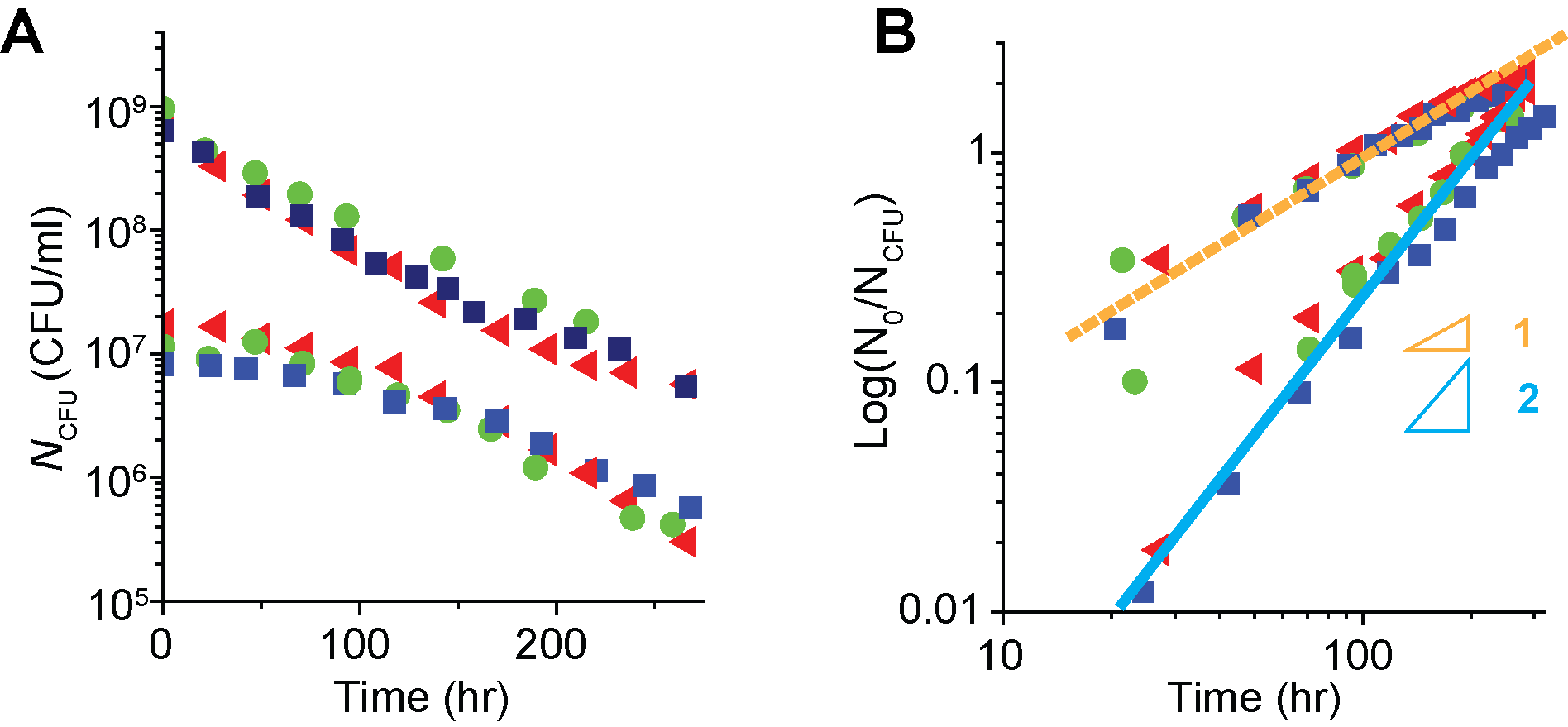

Supplement: S8 Fig — To accurately determine the number of colony forming units, we used serial dilutions and ensured the number to be around 100~200 per agar plate. Also, we had three replicates and reported the average values from the 3 replicate measurements for N CFU (see Materials and methods). Then, we repeated this procedure three times independently and plotted the data in the panel A. In the panel B, we analyzed the data similarly as described in the caption of Fig. 3. The analysis shows an agreement among all three independent experiments; at high cell density the slope is 1, and at low cell density the slope is initially 2. Thus, our data is highly reproducible. (TIF) [file pcbi.1004198.s009.tif]
